# Parametric Production of Prostheses Using the Additive Polymer Manufacturing Technology Multi Jet Fusion

**DOI:** 10.3390/ma17102347

**Published:** 2024-05-15

**Authors:** Karel Ráž, Zdeněk Chval, Vladislav Kemka

**Affiliations:** Faculty of Mechanical Engineering, Regional Technological Institute, University of West Bohemia, Univerzitni 2732/8, 301 00 Plzen, Czech Republic; zdchval@fst.zcu.cz (Z.C.); kemka@fst.zcu.cz (V.K.)

**Keywords:** 3D printing, additive manufacturing, PA12GB, prosthesis, automated design, MJF

## Abstract

This study aims to develop a procedure for the production of 3D-printed forearm prostheses (especially hard outer sockets). The production procedure is designed in the form of a parametric workflow (CAD model), which significantly speeds up the designing process of the prosthesis. This procedure is not fixedly dependent on the software (SW) equipment and is fully transferable into another SW environment. The use of these prostheses will significantly increase the comfort of their patients’ lives. It is possible to produce prostheses faster and in larger amounts and variants by the usage of additive technology. The input for the own production of the prosthesis is a model of the internal soft socket of the patient. This soft socket (soft bed) is made by a qualified prosthetist. A 3D-scanned CAD model is obtained afterward using the scanning method by an automatic laser projector. An editable, parametric external socket (modifiable in any CAD format) is generated from the obtained 3D scan using a special algorithmic model. This socket, after the necessary individual modifications, is transferred to 3D printing technology and produced using powder technology Multi Jet Fusion (HP MJF). The result of the designed and tested procedure is a quickly editable 3D-printed outer socket (main part of prosthesis), which is able to fully replace the current long-fiber composite solution. Production of current solutions is relatively time-consuming, and only one piece is produced in a given time. The newly designed technology eliminates this. This study summarized the possibilities of speeding up the production of forearm prostheses (but not only these) by creating a parametric CAD model that is applicable to different patients.

## 1. Introduction

Manufacturing of prosthetics is a fascinating branch of modern biomedical area and technology that brings significant benefits to patients with amputations or birth defects. There are various reasons why prostheses are made, and these reasons include improving quality of life, restoring functionality, and replacing lost limbs [1,2]. Below are some key reasons for making dentures.

One of the goals of prosthetics is to enable patients who have lost a limb to return to normal life with as much mobility as possible. Modern prostheses are designed to best imitate natural movement and allow patients to carry out normal activities [3].

Aesthetics is another important factor that plays an important role in the psychological and emotional comfort of patients with amputations. The development of prosthetic technologies focuses on creating realistic and aesthetically suitable prostheses that help patients feel more comfortable in society and restore their self-esteem [4,5]. Quality prostheses allow patients to be independent and fully participate in society. The ability to move and perform normal activities without major restrictions can significantly increase the standard of living of an individual with an amputation [6].

There is constant technological progress in the field of prosthesis production. New materials, sensors, and integrated technologies allow the creation of increasingly advanced prostheses with better functionality and adaptability to the needs of specific patients [7]. Each patient has unique needs and requirements. Modern prostheses are designed by taking into account the patient’s individual anatomical and functional characteristics [8]. This achieves the best possible fit between the prosthesis and the patient.

Overall, it can be concluded that the production of prostheses brings many advantages for individuals with amputations, not only from a physical point of view but also psychologically and socially [9,10]. Constant research and innovation in this field are pushing the boundaries of what is possible, contributing to the continuous improvement of the lives of people with amputations.

The research is focused on the use of 3D printing in medical devices, where the emphasis is on the absolute precision of shapes for patient safety. There are various printing techniques (Fused Deposition Modeling, Multi Jet Fusion, etc.) that can be used in this application. It is possible to use 3D-printed composite materials with long fibers (for example, carbon, aramid, or glass fiber ones) or with short fibers (mainly glass ones). The prosthesis is divided into several parts (inner soft socket, outer hard socket, ring for connection to the myoelectrical hand, etc.). The purpose of the designed outer socket is to connect the inner socket with the functional elements of the prosthesis, to protect the stump and the internal mechanical or electrical parts of the prosthesis, and to provide a cosmetic or design replacement with an emphasis on the individual needs of the patient [11,12]. All of these requirements are suitable for the use of new 3D printing technologies, but the exact construction and design process needs to be verified and validated in collaboration with patients [13].

## 2. Conceptual Methods and Objectives of the Prosthesis Development

**Figure 1:**
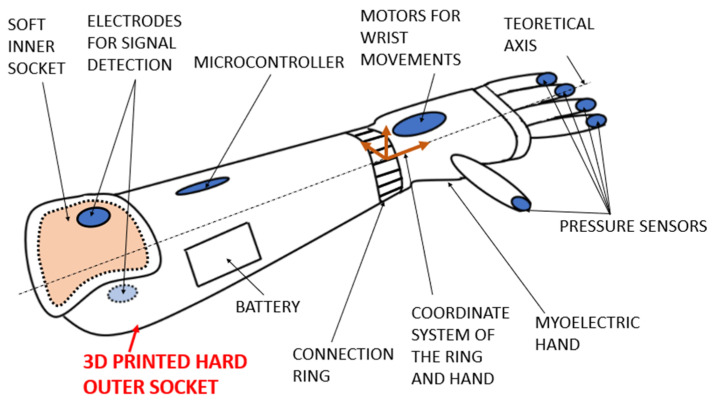
Schematical view of myoelectric forearm prosthesis.

The goal of the design is to create a 3D CAD parametric and modifiable model of the outer socket (also called outer bed or hard socket). This socket will then be fully usable for 3D printing technology, and it will be possible to create a larger number of various sockets (for example, as a spare part, reinforced ones, lightweight ones, etc.) for a given patient in a relatively short time [14]. The goal is to create a CAD model that will be suitable for powder 3D printing technology and will be based on the scanned geometry. The “basic 3D socket model” will be a widely applicable CAD parametric model that can be automatically used to generate a printable forearm prosthesis (Figure 1) [15]. Most of the steps will be parametric and will be based on the basic model obtained by scanning the soft inner socket. Selected parameters, such as the thickness of the socket shell or the position of the ring coordinate system (ring is used for the connection with the wrist), will be controlled using user-defined parameters.

### 2.1. Manufacturing Technology

The MJF 3D printing method used is based on the sintering of the powder material [16]. There are various possible materials; the one used in this research is PA12GB (polyamide with 40% of glass beads) [17]. It is necessary to mention that this 3D-printed outer socket is not in direct contact with human skin. There is still a soft inner socket (made from silicon) for that.

Unlike the laser powder sintering, this 3D printer uses Multi Jet Fusion technology developed by HP. Multi Jet Fusion technology uses the application of a so-called fusion agent to define the shape of the resulting part [18,19]. This helps the absorption of infrared radiation into the plastic powder. A fine layer of powder is applied over the entire printing surface and heated to a temperature close to the melting point [20]. As the print head moves over the print surface, it applies droplets of fusing agent in the desired shape of each part in the given layer to the powder layer. Subsequently, the infrared lamp heats the printing layer [21].

This causes a local increase in temperature above the melting point of the material. The printing surface is then moved down by one layer, and the whole process is repeated.

**Figure 2:**
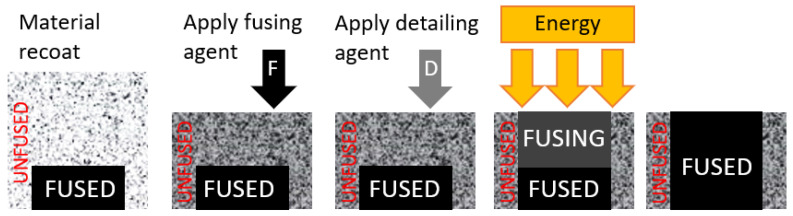
Principle of the MJF technology.

The following Figure 2 shows the process in more detail and graphically. Firstly, the material is deposited on the work surface, then the fusion agent (F) is selectively applied where the powder particles are to be joined together. Afterward, the detailing agent (D) is selectively applied where the fixing effect needs to be reduced or enhanced. In this example, the detailing agent will limit the melting to the boundary and create a part with sharp and smooth edges. The work surface is exposed to melting energy during the further step. Finally, a layer of bonded material is created at the point where the fusion agent is applied. The process is repeated until the entire part is created [22,23,24].

### 2.2. Material Description

**Figure 3:**
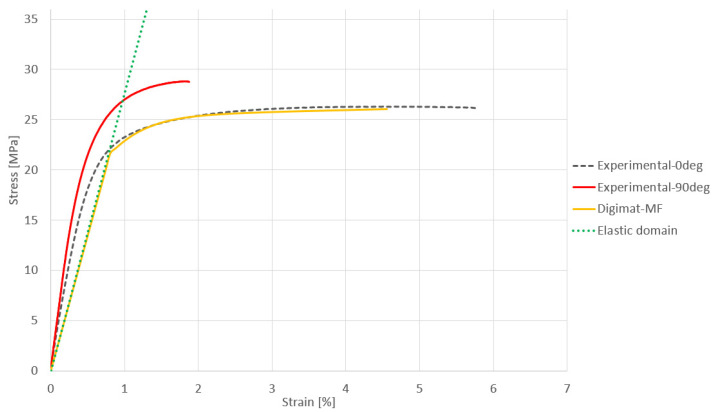
Stress–strain curves for PA12GB.

The thickness of one layer for the material PA12GB is around 0.08 mm, and this technology results in an actual productivity of 4115 cm^3^/h [25,26]. The processing temperature for the PA12GB is around 160 °C to 180 °C [27]. This temperature has to be understood with respect to the powder melting point of PA12GB, which is 186 °C [28]. The material PA12GB has a tensile strength of 30 MPa (according to the official datasheet) [29]. The guaranteed value of elongation at brake, given by the producer of the material, is 6.5% (see the following Figure 3) [30]. It is obvious that the material properties are more complex compared to the official datasheet, as is shown in the following figure. It shows the effect of the orientation during the printing process.

These results were obtained during material testing with respect to the regularly used standard.

The gray dashed curve represents the stress–strain curve of PA12GB at a 0-degree angle, while the red curve represents the curve for PA12GB at a 90-degree angle. The difference is observed in the plastic domain, where failure occurs much earlier for the 90° test compared to the 0° test.

### 2.3. Functional Model of the Soft Inner Socket of the Prosthessis

**Figure 4:**
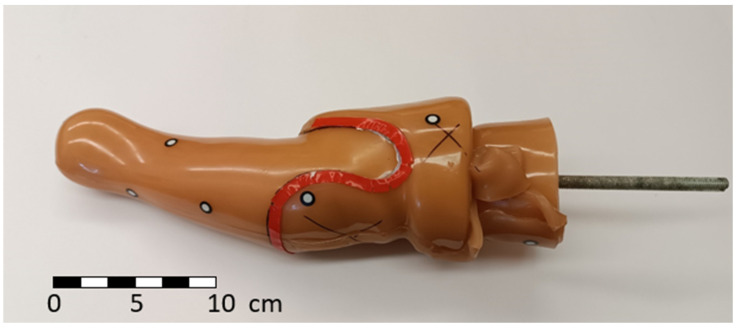
Input geometry for the CAD model of the inner socket (inner socket).

The model of the soft inner socket of the forearm prosthesis was used as an input for creating a model for 3D printing. This model was created in a specialized prosthetics workplace, and shape adjustments (for example, anatomical reductions for better attachment [31,32]) were made to it. In the following Figure 4, the shape of the forearm socket itself is obvious (the socket is ready for scanning).

### 2.4. Inner Socket Scanning

The scanned object was placed in a vertical position on a flat base, and targets were placed on its surface. Their distances between points with respect to the recommendations were in the ranges of 150 to 250 mm [33]. The typical arrangement of targets is obvious from the previous picture.

The measurement was carried out in a laboratory with a controlled environment with a temperature of 20 ± 1.0 °C and a relative humidity of 50%. The system (scanning head) was calibrated on the calibration board supplied by the manufacturer using the calibration procedure within the software.

The accuracy of the scan was set by a resolution of 0.2 mm [34]. Because the prosthesis is made of silicone and, therefore, of a material that greatly absorbs the laser scanning beam, a higher beam intensity (shutter) was set (value 5.4) [35,36].

Firstly, only the retroreflective target positions were scanned, a positioning model (PM) was created, and a coordinate system was defined. After that, the laser scanner system was activated in the mode of seven laser lines [37], and the surface of the object (cloud of points) was scanned by smooth movement of the scanner around the entire object.

The point cloud was converted to a mesh model without major subsequent mesh modifications, and the resulting mesh was exported to an stl file in the VXelements software (version 11) [38,39].

The VXelements 3D scanner utility is used to process point clouds from the scanning head. It is also advisable to take into account the recommended configuration of the computer because the HandySCAN 3D scanner collects a large amount of data (especially when using a higher resolution), and it is necessary that the computer is able to process these data in an acceptable time [40,41].

### 2.5. Scanning Device

**Figure 5:**
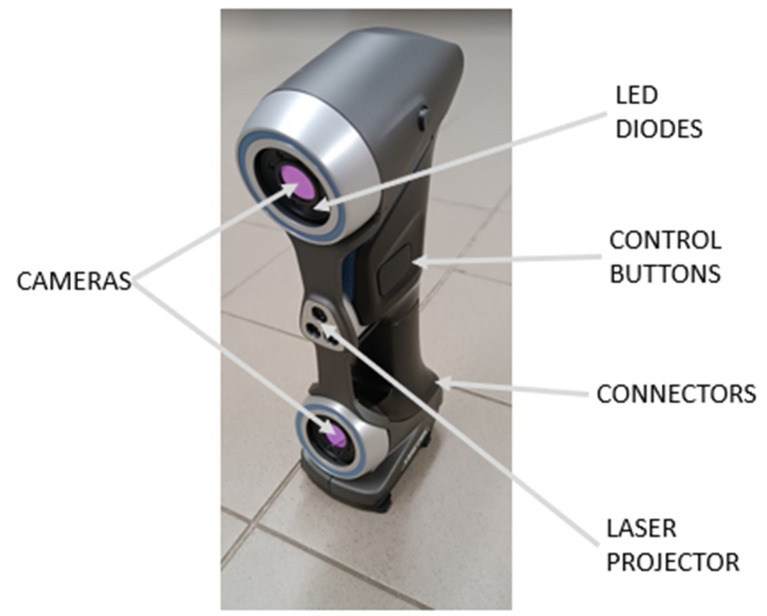
Scanning device HandySCAN 307.

HandySCAN 307TM (Figure 5) is a portable 3D system (scanner) manufactured by Creaform company, (Irvine, CA, USA). The system uses retro-reflective targets to orient the scanning head in a spatial coordinate system and uses a laser projector with multiple laser lines to scan the surface of objects based on triangulation [42]. Two cameras located at opposite ends of the scanner detect both retro-reflective targets (hereinafter referred to as targets) and projected laser lines [43]. The location of the targets is used to create a position model (PM) that orients the scanner in space and defines the appropriate coordinate system. The output is usually a set of (x, y, z) coordinates in a Cartesian coordinate system. Most 3D imaging systems are based on three measurement techniques, i.e., interferometry, triangulation, and beam time of flight [44,45,46].

### 2.6. Parameters of the Scanning Method

The applied method for scanning within the research has the following specific parameters (Table 1) [47].

**Figure 6:**
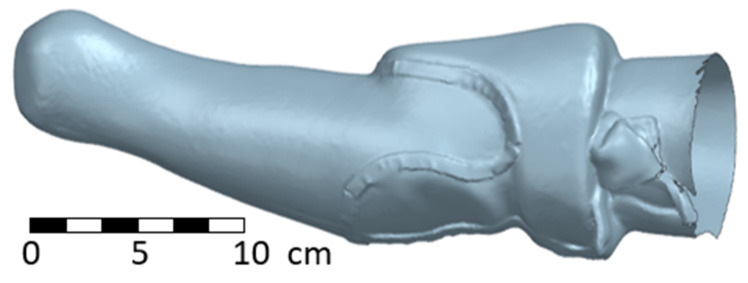
Scan of the input geometry for the forearm socket—side view.

**Figure 7:**
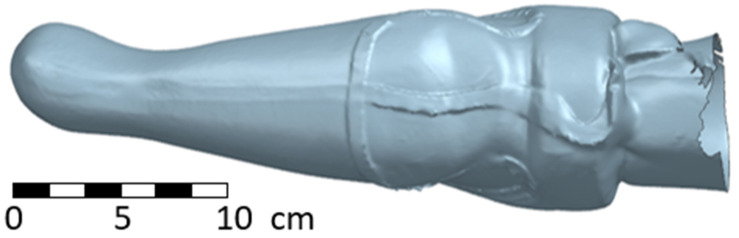
Scan of the input geometry for the forearm socket—frontal view.

The resulting stl geometry of the inner soft socket is obvious in the following two figures (Figure 6 and Figure 7).

## 3. Parametric Workflow of the Prosthesis Workflow Generation

The aim of the research is to create a CAD model that will be as universal as possible for different patients and will be based on the obtained scans.

**Figure 8:**
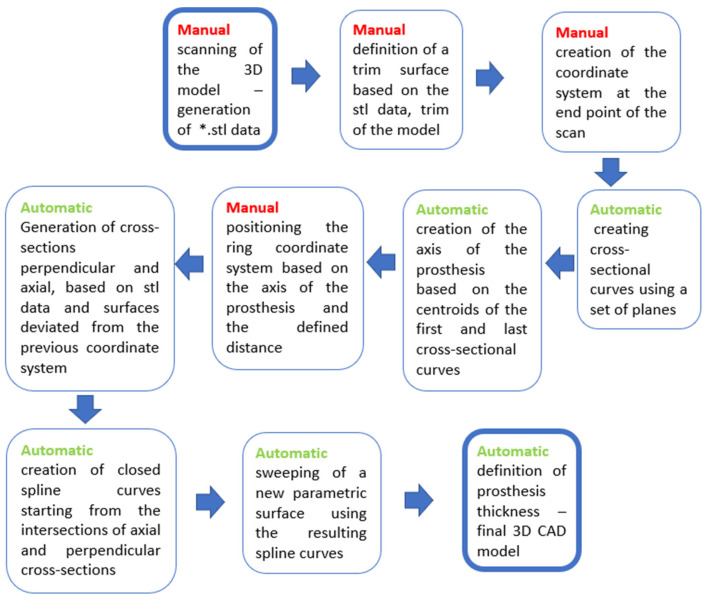
Parametric workflow of prosthesis creation.

For this reason, a workflow describing individual steps (Figure 8) from the actual scanning to the final prosthesis model was created. Automatic and manual steps are separated by color in the actual workflow (manual steps are in red; automatic steps are in green color).

**Figure 9:**
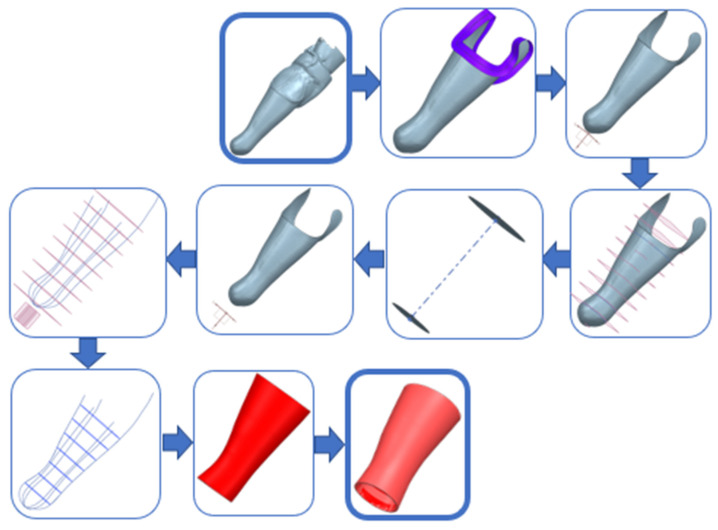
Parametric workflow of prosthesis creation—graphical CAD representation.

The same unique procedure can be shown in the form of pictures (Figure 9) of individual steps, as is shown in the following figure.

**Figure 10:**
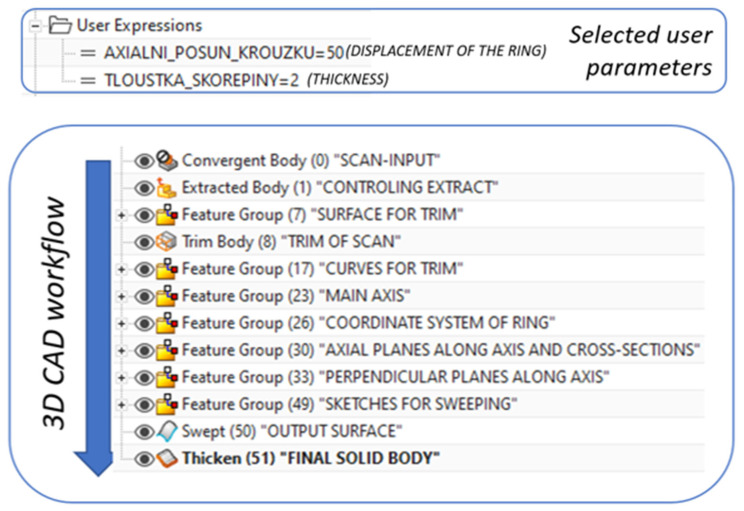
Example of workflow within the CAD software with specified user parameters.

A parametric CAD model is used as part of the automatic generation of the prosthesis model for 3D printing. This is defined using two user parameters (displacement of the center of the connection ring of the prosthesis and the thickness of the prosthesis). The part history (defined by features) within the various CAD software is divided into functional blocks, as can be seen from the following diagram (Figure 10).

### 3.1. Comparison of the Orginal Scan with the Automaticaly Generated Surface

For the correct functionality of the outer socket, it is necessary to ensure maximum correspondence between the original and the newly generated surface.

**Figure 11:**
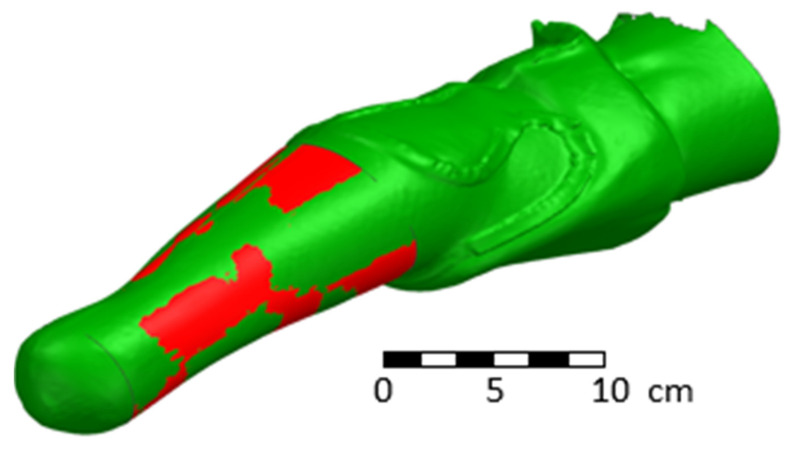
Overlay of the original scan (green color) and the generated parametric inner surface of the socket (red color).

It is possible to allow minor differences. These differences should not be more than 1 mm, as determined by consultation with qualified prosthetists [48,49,50]. Both geometries are shown in the following Figure 11. The design area and overlaying of the original scanned surface (green) and newly designed area (red) are obvious. The same differences were obtained from the 3D printing technology.

**Figure 12:**
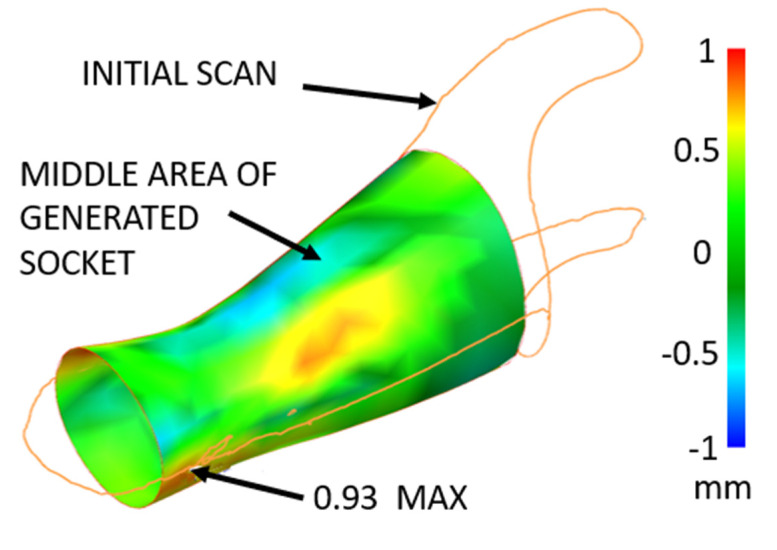
The maximum deviation between the scanned surface and the generated surface.

The next step is to perform an analysis of the differences between the two surfaces. It is clear that the maximum value of the difference reaches 0.93 mm, which is fully acceptable from a functional point of view (see Figure 12).

### 3.2. Production and Experimental Testing

**Figure 13:**
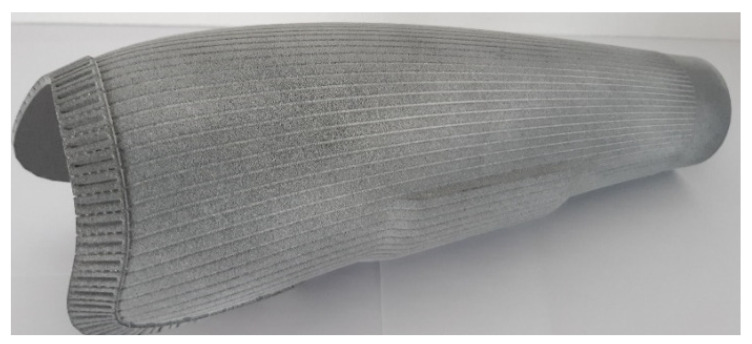
3D-printed outer socket from PA12GB.

The last step before handing over the prosthesis to the patient is its printing using the PA12GB material (supplier Hewlett-Packard, Palo Alto, CA, USA). The printed outer socket is shown in the following Figure 13.

The prosthesis meets all requirements, and it is fully comparable to the original composite laminated solution. A great advantage of this printing is the possibility of arbitrary surface treatment, as different coatings can be easily applied to this polymer material [51,52].

## 4. Conclusions

This article summarizes the possibilities of creating a parametric model of the outer socket of a 3D-printed prosthesis. This parametric CAD model is based on a scan of the internal socket created by a qualified prosthetist. Based on this scan, an external socket is automatically generated. This procedure is applicable to different patients. With this procedure, the delivery time of the prosthesis is significantly accelerated, as the original lamination technology is replaced by 3D printing technology. Using additive manufacturing, it is possible to easily produce a larger number of prostheses (for example, for different purposes during daily activities).

As part of the research, a parametric workflow is precisely described, which is transferable to different patients after hand amputation. It has been verified that the shape of the original and generated surface is within the required tolerance. The automatically generated area of the outer socket is created by the sweep command, and it is a fully parametric CAD model that offers a wide range of adjustment options according to the patient’s requirements.

As part of the testing of the proposed method, the functionality of the 3D-printed prosthesis was verified. Further research will be focused on testing other materials and creating a database of design looks. The patient will then have the opportunity to choose the appearance or color directly and will be supplied with a corresponding 3D-printed prosthesis.

## Figures and Tables

**Table 1:** Parameters of the scanning process.

Accuracy	up to 0.040 mm
Volumetric accuracy:	0.020 mm + 0.100 mm/m
Measuring resolution:	0.100 mm
Resolution of the net:	0.200 mm
Sampling rate:	480,000
Laser beam:	7
Laser class:	2 M
Scanned area:	275 × 250 mm
Distance from the scanned object:	300 mm
Depth of sharpness:	250 mm
Maximal size of scanned object:	0.1–4 m
Software:	VXelements

## Data Availability

Not applicable.
